# Multi‐site Ultrasound‐guided Fine Needle Aspiration to Study Cells and Soluble Factors From Human Lymph Nodes

**DOI:** 10.1002/cpz1.70063

**Published:** 2024-11-23

**Authors:** Adam Al‐Diwani, Deepsha Agrawal, Fintan Sheerin, Callum Board, Sarosh R. Irani, Katrina M. Pollock, Nicholas M. Provine

**Affiliations:** ^1^ Department of Psychiatry University of Oxford Oxford UK; ^2^ Department of Radiology, John Radcliffe Hospital Oxford University Hospitals NHS Foundation Trust Oxford UK; ^3^ Oxford Autoimmune Neurology Group, Nuffield Department of Clinical Neurosciences University of Oxford Oxford UK; ^4^ Departments of Neurology and Neurosciences Mayo Clinic Jacksonville Florida; ^5^ Oxford Vaccine Group, Department of Paediatrics University of Oxford Oxford UK; ^6^ Pandemic Sciences Institute, Nuffield Department of Medicine University of Oxford Oxford UK; ^7^ Centre for Human Genetics, Nuffield Department of Medicine University of Oxford Oxford UK

**Keywords:** cervical lymph nodes, fine needle aspiration, lymph, lymph node

## Abstract

Lymph nodes (LNs) are specialized secondary lymphoid tissues essential to the priming and maintenance of adaptive immune responses, including the B cell germinal center response; thus, they are central to immunity. However, the anatomically restricted and time‐resolved nature of immune priming means that sampling disease‐relevant human LNs requires specialized techniques. This article describes the application of ultrasound‐guided fine‐needle aspiration (FNA) to sample LNs, using cervical LNs of the head and neck as an exemplar. This minimally invasive technique allows collection of both immune cells and cell‐free material that are relevant to both neuroimmune diseases and basic lymphatic functions. Downstream use of cellular material can include multiplexed flow cytometry, single‐cell transcriptome sequencing (RNA‐seq), and B cell cultures. The cell‐free supernatant can be used for proteomics or other similar ‘omics approaches. This unit describes collection of samples by FNA as well as processing and storage of samples for downstream assays. © 2024 The Author(s). Current Protocols published by Wiley Periodicals LLC.

**Basic Protocol 1**: Sampling of human cervical lymph nodes by ultrasound‐guided fine‐needle aspiration

**Alternate Protocol**: Sampling of human lymph nodes by ultrasound‐guided fine‐needle aspiration with negative pressure

**Basic Protocol 2**: Processing and storage of human lymph node samples

## INTRODUCTION

Lymph nodes (LNs) are secondary lymphoid organs that receive drained tissue fluid via afferent lymphatics. This contains soluble macromolecules and migratory immune cells, and in the context of infection may also include microbes. Adapted to surveil the drained tissue, LNs bring these potential antigens into close apposition with antigen‐presenting cells and circulating and resident lymphocytes. This architecture is adapted to support the priming of naive T cells and B cells, as well as the germinal center reactions that drive honed immune memory towards specific antigenic targets. Given the centrality of LNs to the initiation of adaptive immune responses, samples collected from them are likely to be of fundamental relevance to every immune‐associated process, including infection biology, autoimmunity, vaccinology, and even neuroscience. Thus, their safe and effective sampling in human participants to study both cellular and fluid‐phase components is a valuable endeavor.

Immune reactions in the lymph node are spatially and temporally resolved, at both the tissue and whole‐organism levels. In the context of an acute exposure (e.g., infection), priming of T cells by interaction with activated dendritic cells expressing major histocompatibility complex (MHC)‐loaded cognate antigen occurs within days (Williams & Bevan, 2007). Once T cells are successfully primed, the effector cells exit the LNs and home to affected tissues. In most acute settings, B cell activation begins as an extrafollicular response, which is augmented by the germinal center response as specialized T cell help becomes sufficient to sustain the process (Elsner & Shlomchik, 2020). The germinal center response is the primary contributor to both memory B cell and long‐lived antibody‐secreting plasma cell populations (Victora & Nussenzweig, 2022). Although the germinal center response can persist for months after a primary exposure (Turner et al., 2021), there are clear changes in the composition of the germinal center with time, with some models suggesting a temporal switch from memory B cell to plasma cell output (Weisel et al., 2016). In the context of chronic conditions, these dynamics become more complicated because of antigen re‐exposure, but immune profiles are substantially affected by specific disease states, including remission and flares, and therapeutic interventions. For example, we found that rituximab treatment has a major impact on B cell frequency and autoantibody concentrations specifically within the LN (Damato et al., 2022). Collectively, this illustrates the complex immune dynamics and isolated environment within the LN, which must be accounted for when designing experiments to study this tissue or its downstream effects.

Anatomy must also be considered. LNs are distributed throughout the body, and specific LNs drain the afferent lymphatics of specific tissues. For example, the axillary LNs, located in the armpits, drain the upper arms and are thus relevant for studying vaccination following intramuscular vaccination in the deltoid muscle (O'Rahilly et al., 2008). In contrast, the cervical LNs (CLNs), located in the neck, are thought to collect afferent lymph from the head and neck, via cerebrospinal fluid efflux into meningeal and then cervical lymphatics (Eide et al., 2018; Louveau et al., 2015). Thus, when studying an immune response in the context of a specific disease, it is necessary to sample the relevant LN. Given this, lymphoid tissue that is commonly removed surgically (e.g., tonsils, cystic LNs during gallbladder surgery, or axillary LNs during breast cancer surgery) is anatomically relevant only in the context of specific disease processes. Thus, there is a critical need for a sampling technique that can target other disease‐relevant LNs. Ultrasound‐guided LN fine‐needle aspiration (LN FNA) is a very suitable sampling approach to address these practical requirements.

Beyond immune cells, lymph and LN‐associated fluids are also rich sources of information about both the afferent tissues and the local immune response. Direct cannulation of the lymphatics in mice has demonstrated the tissue‐specific nature of the proteins detected (Nanaware et al., 2024). Analysis of human lymph and plasma also reveals unique features of the proteome based on sample source (Broggi et al., 2019; Clement et al., 2010, 2013; Nanaware et al., 2024), suggesting that unique insights can be gained by such analysis. We have recently demonstrated that the cell‐free supernatant collected from the CLN is enriched in both CNS‐relevant proteins and immune‐related proteins (Al‐Diwani et al., 2024; Provine et al., 2024). Thus, it is possible to directly sample LN tissue and generate data on both the immune cells and LN‐associated soluble analytes.

Here, we describe the application of FNA under ultrasound guidance to sample human LNs, with cervical LN sampling used as an exemplar. FNA is a commonplace physical investigation in various medical contexts, including pathology involving LNs, but driven by advances in downstream ‘‐omic’ technologies, the technique has in recent years been shown to be highly adaptable to healthy LNs in a clinical research context and to address the temporal and anatomical considerations discussed above. As an additional strength, supernatant from resuspension of the aspirated LN tissue can also be analyzed, a parameter not considered in previously published protocols (Bettini et al., 2022; Havenar‐Daughton et al., 2020). Given its minimally invasive nature, the technique is also suitable for repeated sampling. In these protocols, we showcase, from participant to bench, two approaches that we have used for LN sampling. These are optimized either for more efficient sampling of the extracellular lymphatic fluid or for increasing cellular recovery, dependent on the scientific need.

*CAUTION*: All necessary ethical approvals must be in place before beginning work involving human subjects. Informed consent must be obtained and appropriately recorded for all participants. All relevant laws and institutional guidelines relating to research involving human participants must be followed. For our work, all samples were obtained with informed consent in accordance with the 1964 Declaration of Helsinki and ethical approval (NHS Research Ethics Committees 16/YH/0013 and 23/LO/0459).

*CAUTION*: This protocol involves human material; thus, all standard blood‐borne pathogen (BBP) safety protocols for your institute must be followed.

*CAUTION*: This protocol is a sterile procedure and should be done under standard sterile technique. Ultrasound gel can be used but minimal contact with the aspirated cellular material is encouraged.

*CAUTION*: Historically, Basic Protocol 1 has been used extensively for cervical lymph node sampling of the head and neck, while Alternate Protocol has been used extensively for sampling of the axillary lymph nodes. Consultation with radiologists should be undertaken before the application of either sampling protocol to the specific patient population and lymph nodes of interest, as specific clinical considerations may be present.

## Basic Protocol 1 SAMPLING OF HUMAN CERVICAL LYMPH NODES BY ULTRASOUND‐GUIDED FINE‐NEEDLE ASPIRATION

**Figure 1:**
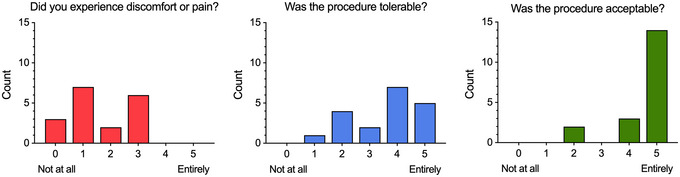
Participant satisfaction survey. Participant survey data (*n* = 19) on experience of CLN FNA. Survey participant age range from 26 to 75 years.

Accessing human cervical lymph nodes (CLN) provides biological material relevant to general LN function (Provine et al., 2024) but specifically, through their anatomical links with the meningeal lymphatics, CLNs are also relevant to neuroscience research (Al‐Diwani et al., 2022, 2024; Eide et al., 2018; Louveau et al., 2015; Nanaware et al., 2024; Provine et al., 2024). We have sampled (*n* = 44) human CLNs using FNA for research purposes over the past 7 years across several neurologic and neuropsychiatric cohorts, with high levels of participant‐reported acceptability and no adverse events across a range of participant ages (19‐78 years of age) (Fig. 1). Central to this safety record has been procedure delivery by senior head and neck radiology colleagues. Expertise in complex neck anatomy together with imaging guidance allows precise targeting and minimization of risk to other structures. Consequently, participants can be counselled that, under these conditions, the procedure risk is no greater than for a blood test. Given the present, albeit low, risk to the participant, we currently only perform this procedure on participants who have the mental capacity to consent to the procedure for themselves. Given that we have historically used this protocol for CLN sampling, consultations should be made before application to sampling of other LNs.

Briefly, the participant is asked to give consent for the procedure. Then, the radiologist uses an ultrasound probe to identify a CLN that is appropriate for sampling (e.g., not too deep, easy to visualize, not too close to major arteries). Then, using ultrasound guidance, a small needle is inserted into the CLN and moved around to draw LN material into the needle. LN material is then collected from the needle for downstream applications. The whole procedure is repeated once to increase LN material recovery.

**Figure 2:**
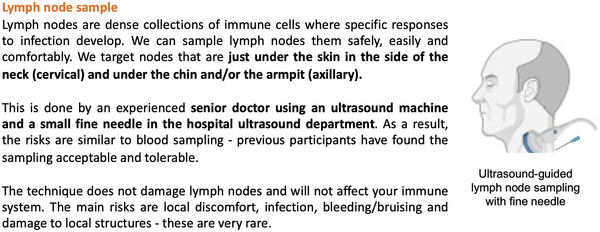
Example informal participant information. Example text used in the informal participant information document as part of ANIMATE study (23/LO/0459) to discuss LN FNA.

We provide examples of both informal and formal information provided to prospective participants in studies involving LN FNA:

- An example informal participant information form is presented in Figure 2.
- An example of a participant information sheet for a study that includes LN FNA can be found at https://trials.ovg.ox.ac.uk/trials/legacy03
- An example participant information video for the same study can be found at https://www.ovg.ox.ac.uk/news/ground‐breaking‐research‐revealed‐in‐new‐video

### Materials

Participant information leaflet and video of procedure
Ultrasound machine and probe (GE Healthcare LOGIQ E10, or equivalent)
Drape
Sterile wipes
Sterile sleeves for ultrasound probe
Sterile 23‐G (blue) needles
5‐ml syringe
Eight 1.5‐ml Eppendorf tubes, each pre‐filled with 1 ml sterile Dulbecco's phosphate‐buffered saline (D‐PBS)

### Patient consent and preliminary preparations

1 Inform the potential participant about the rationale for CLN sampling, the main steps involved, and the risks, together with how these are minimized. Give the participant time to learn more, including perusing video and written resources, and to ask further questions. If they wish to proceed, have them complete the study‐specific informed consent form.
2 Pre‐fill eight 1.5‐ml microcentrifuge tubes with 1 ml each sterile D‐PBS.
3 On the day of the procedure, the participant, researcher, and radiologist should meet at the ultrasound suite.
  This is a specific room that includes the ultrasound machine and a reclinable hospital bed.

### Sampling of the lymph node (by radiologist)

**Figure 3:**
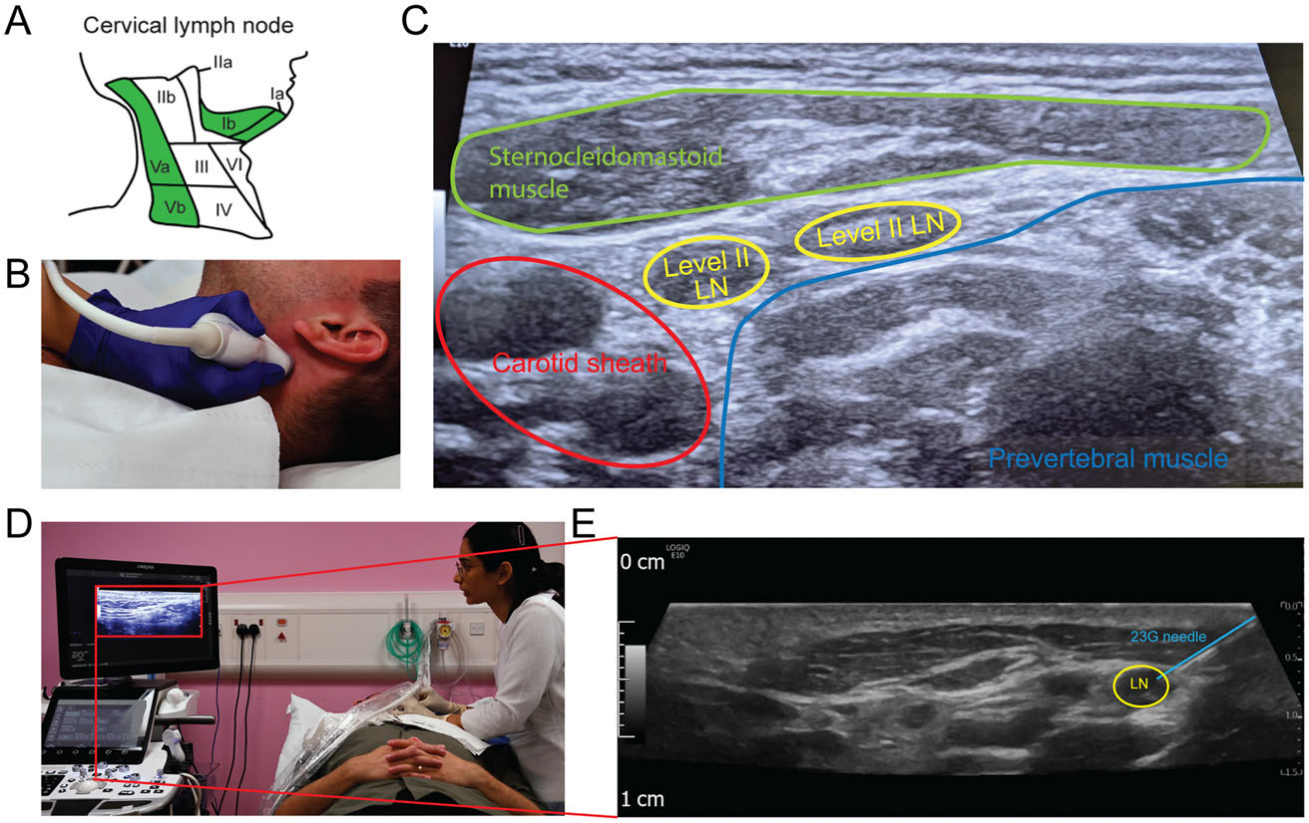
Cervical LN sampling. (**A**) Anatomic localization of the cervical LNs within the neck. Our prior sampling has been primarily from level 1 and 5 LNs, highlighted in green. Reproduced from Al‐Diwani et al. (2022). (**B**) Locating an accessible cervical LN. (**C**) Ultrasound image of the neck with detailed anatomic annotation of key major structures. (**D**) Sampling of the cervical LN under ultrasound guidance. (**E**) Ultrasound image of the neck with the LN and the FNA needle highlighted.

4 Go through the procedure checklist, which should include checking participant understanding, verbal procedural consent, and reviewing medications to consider bleeding risk.
  Perform welfare checks throughout the procedure, e.g., during step 9 and after step 10.
5 With the participant positioned at an angle of ∼15 degrees, with a pillow for head support, ultrasonographically inspect CLN levels I, II, III, and V for fine‐needle accessibility and proximity to vessels using doppler (Fig. 3A‐C).
  Often, only a single LN is easily accessible for sampling for a given participant.

6 Once a target level and node have been selected, sterilize the overlying skin using a sterile wipe.
7 Don sterile gloves, and place a sterile sleeve over the ultrasound probes and cable.
8 After further visualization, unsheathe a sterile 23‐G blue needle. Holding the needle between your fingers, insert it through the skin into the target node under direct ultrasound visualization (Fig. 3D and E).
  The needle bevel should be held directly between the fingers, and no syringe should be used.
9 Under ongoing ultrasound guidance, manually move the needle tip up and down in a twisting rotary corkscrew motion in the lymph node, at a rate of about two up‐and‐down motions per second. This causes the needle tip to move more shallowly and more deeply in the lymph node.
  For reference, the way this approach is described technically by one radiologist is: The needle tip is moved rapidly shallower and deeper along its long axis within the node with a frequency of ∼2 Hz. An additional rotary motion is applied to the needle bevel tip.
  Note that in this approach, no external negative pressure is applied—the material enters the needle bevel through capillary action.
10 Remove the needle and place it on a clean surface to be transferred to the researcher.

### Sample processing (by researcher)

11 Attach a 5‐ml syringe to the needle. Place the needle tip in the first of the 1.5‐ml microcentrifuge tubes containing D‐PBS, and pull up 1 ml of the D‐PBS through the needle into the syringe.
12 Remove the syringe and replace the diluted aspirate material in the corresponding tube. Cap the tube and place on ice.
  The samples can be stored on ice for the duration of the procedure, but should be processed as soon as possible upon procedure completion, as described in Basic Protocol 2.
13 Repeated steps 11 and 12 for a total of four washes, collecting the wash in a separate microcentrifuge tube each time.
14 Dispose of the needle in an appropriate sharps container.

### Resampling of lymph node (by radiologist)

15 Meanwhile, target the same LN with a fresh needle and repeat steps 8‐14 with this second needle.
16 Further clean the access point, and apply a small plaster or dressing.
  The dressing can be removed by the participant later in the day.
17 Allow the participant to sit up gradually, and perform a welfare check.
  In our experience, these two passes are sufficient and are tolerated well by participants. Exceptionally, more passes can be done if further cell retrieval is critical, but this would need discussion with the participant and further ongoing checking of participant welfare. The Alternate Protocol could also be considered.

## SAMPLING OF HUMAN LYMPH NODES BY ULTRASOUND‐GUIDED FINE‐NEEDLE ASPIRATION WITH NEGATIVE PRESSURE

Tissue sampling via Basic Protocol 1 is an efficient for the recovery of LN cellular material while also minimally diluting the recovered sample to preserve detection of soluble analytes within the supernatant. However, in some applications, increased cell yield is desired for downstream applications. Inclusion of negative pressure during aspiration results in an approximately threefold increase in cell yield (Havenar‐Daughton et al., 2020). Because of the sample dilution steps in the cell recovery procedure, however, this does decrease sensitivity for the measurement of LN‐associated soluble factors. This protocol describes how to perform LN sampling using negative pressure with a focus on maximizing recovery of LN cells.

This protocol has used successfully by us (Day et al., 2022; Siu et al., 2024) and others (Havenar‐Daughton et al., 2020; Lederer et al., 2022; Turner et al., 2020) for sampling the axillary LNs. Consultations should be made before applying the protocol to the sampling of other LNs.

### Additional Materials (also see Basic Protocol 1)

2% (w/v) lidocaine hydrochloride
R10 medium

Five 21‐G (green) needles, sterile
Five 5‐ml syringes
Five 15‐ml conical tubes

### Patient consent and preliminary setup

1 Set up the participant for ultrasound scanning as described in Basic Protocol 1, steps 1‐7. The participant may lie flat with a pillow for support.
2 Don sterile gloves, and place a sterile sleeve over the ultrasound probes and cable. Assemble five 5‐ml syringes and 21‐G needles and place on the sterile trolley.
  Smaller‐gauge needles can also be used
3 If using local anesthetic (e.g., 2% [w/v] lidocaine hydrochloride), infiltrate into skin and soft tissue surrounding LN according to standard dosing and clinical practice guidelines.
  Use of local anesthetic should be by a trained practitioner in accordance with local and national safety guidance for its use. Perform welfare checks during steps 3‐5 and before step 7.

### Sampling of the lymph node (by radiologist)

4 Once the anesthetic has taken effect, holding the syringe between their fingers, insert the needle through the skin into the LN under direct ultrasound guidance as described in Basic Protocol 1, step 8, except that because the needle is attached to a syringe, the syringe body (rather than the needle bevel) may be held.
5 Aspirate fully into the syringe, and manually move the syringe up and down so that the needle tip is moving more shallowly and deeply in the lymph node to aspirate LN cells into the needle (see Basic Protocol 1, step 9).
  Aspiration creates negative pressure, which draws cellular material into the needle body. Because the LN material is highly viscous, the syringe will not fill with liquid. The negative pressure simply helps draw material into the needle and possibly into the syringe body.
  If a blood vessel is accidentally contacted, blood will immediately enter the syringe. This indicates that the procedure has not been correctly performed and the needle should be withdrawn. This does not pose a risk to the patient as it is functionally the equivalent of a veinous blood draw. A fresh needle and syringe should be assembled and, after confirming that the participant still wants to continue, resampling of the same LN can be attempted.
6 Expel the LN cells into a 15‐ml conical tube containing 10 ml R10 medium.
  The larger flushing volume improves cell recovery. If you are interested in quantifying cell‐free material, sterile PBS should be used as the collection medium to minimize background.
  The samples can be stored on ice for the duration of the procedure, but should be processed as soon as possible upon procedure completion, as described in Basic Protocol 2.
7 Meanwhile, target the same node with a fresh needle and perform steps 4‐6 several times, typically up to five times to yield sufficient cells.
8 Clean the access point further and apply a small plaster or dressing.
  The dressing can be removed by the participant later in the day.
9 Allow the participant to sit up gradually and perform a welfare check.

## Basic Protocol 2 PROCESSING AND STORAGE OF LYMPH NODE SAMPLES

In this protocol, we isolate both the cell‐free aspirate supernatant for use in appropriate assays (e.g., proteomic analysis) and the cellular fraction for long‐term storage or immediate use in appropriate assays (e.g., flow cytometry or single‐cell RNA‐seq).

*NOTE*: All work in this protocol should be performed in a tissue culture cabinet to maintain sample sterility.

### Materials

Lymph node aspirates (Basic Protocol 1 or Alternate Protocol)
R10 medium (see recipe)
Ficoll‐paque (1.077 g/ml; StemCell cat. no. 07851 or equivalent)
Trypan blue solution
Fetal bovine serum (FBS), heat inactivated
Dimethyl sulfoxide (DMSO)

Microcentrifuge for 1.5‐ml microcentrifuge tubes
1.5‐ml Protein LoBind microcentrifuge tubes (Eppendorf cat. no. 0030108094)
10‐, 200‐, and 1000‐µl (P10, P200, P1000) pipets and tips
CoolCell LX (or equivalent cell cryopreservation technology)
Dry ice
1.8‐ml cryovials
15‐ and 50‐ml conical tube
Centrifuge with buckets for 15‐ml conical tubes and 5‐ml FACS tubes
5‐ml polystyrene FACS tubes
150‐mm glass Pasteur pipets (VWR cat. no. 612‐1701 or equivalent)
Hemocytometer slide

**Figure 4:**
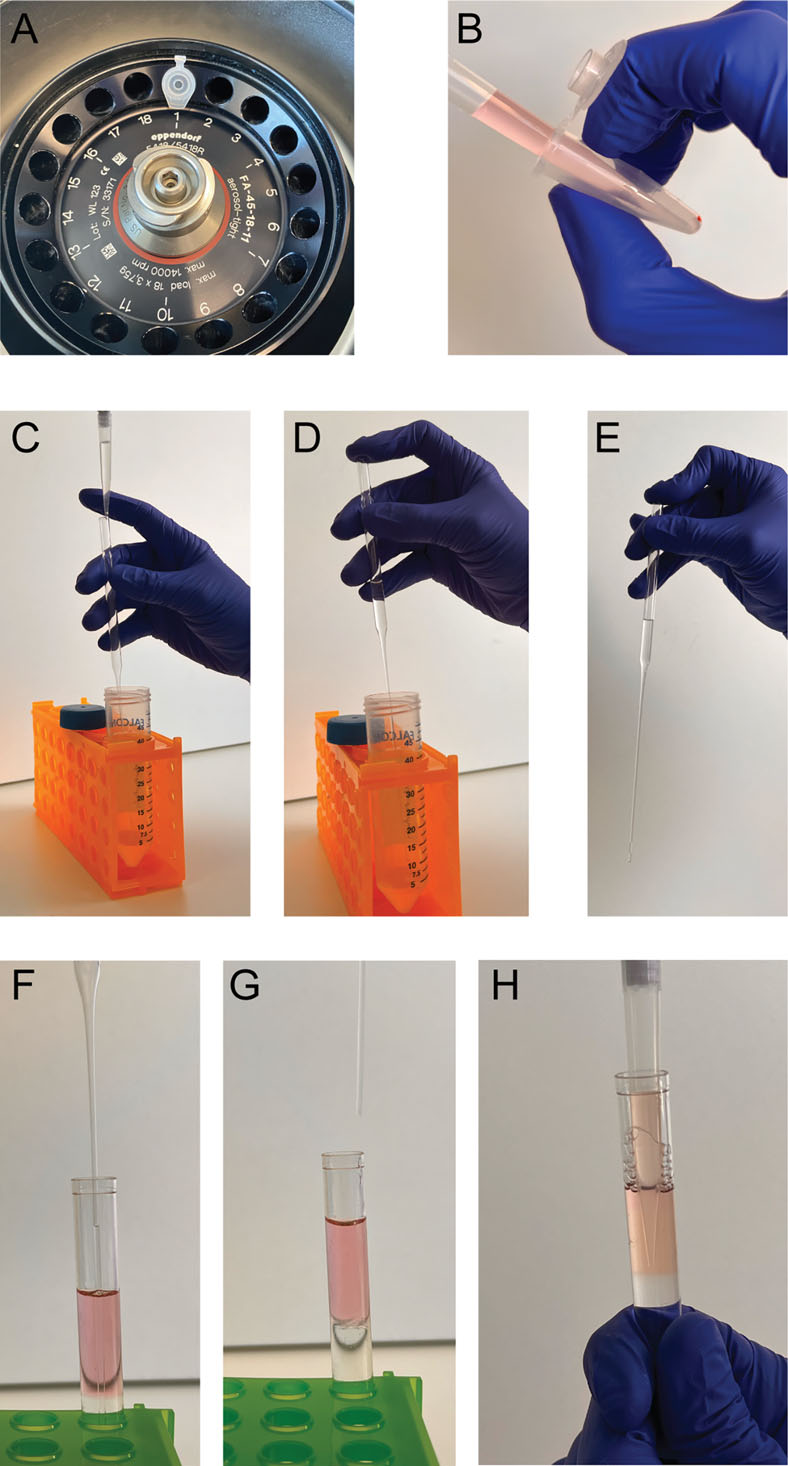
Underlay procedure to remove red blood cells. (**A**) Orientation of sample collection tube from Basic Protocol 1 in the microcentrifuge to aid cell isolation. (**B**) Pipetting technique to remove LN FNA supernatant without disturbing the cell LN cell pellet. (**C**) Filling the Pasteur pipet with Ficoll. (**D, E**) Making a seal on the Pasteur pipet to allow insertion of the pipet into the sample tube. (**F**) Gravity‐aided flow of Ficoll into the bottom of the sample tube, creating the interface. (**G**) Removal of the Pasteur pipet once all Ficoll has drained. (**H**) After centrifugation, pipetting at the medium/Ficoll interface to collect the LN cells.

1 Take all the 1.5‐ml microcentrifuge tubes containing each FNA wash and place in a tabletop centrifuge. Align the tubes so that the hinge on the lid faces upwards (Fig. 4A). Centrifuge 5 min at 400 × *g*, 4°C, in a tabletop microcentrifuge.
  This step pellets the LN cells and allows the supernatant and cellular material to be separated for downstream steps. Orienting the tubes in this way makes it easier to avoid aspirating the cell pellet (next step) even if it is not visible.
  If the Alternate Protocol (FNA with aspiration) is used, this centrifugation step should be performed using a larger‐volume centrifuge to accommodate the 15‐ml conical tubes used to collect the samples.

2 Carefully pipet out the supernatant (∼1 ml volume for supernatant from Basic Protocol 1) without disturbing the cell pellet. Angle the pipet tip so that it does not touch the cell pellet (Fig. 4B). Transfer the supernatant into a new labeled, sterile 1.5‐ml Protein LoBind microcentrifuge tube for storage. Do not merge supernatants from multiple washes. Immediately place on dry ice to flash freeze.
  - Transfer the isolated supernatant to −80°C for long‐term storage, and record in freezer logs, as appropriate.
    This step preserves the supernatant for downstream analysis. Use of Protein LoBind tubes is important as different materials of microcentrifuge tubes can affect protein recovery (Schvartz et al., 2023).
    The supernatants from different washes should not be merged because we have found that they vary in protein concentration, so pooling will result in dilution. We found that the first wash of the second FNA pass (‘needle 2’) had the best protein yield (Al‐Diwani et al., 2024; Provine et al., 2024), so if there is a desire to minimize sample storage, this would be the priority sample.
    To avoid repeated freeze‐thaw, the supernatants can be aliquoted into multiple smaller vials (e.g., five 200‐µl vials) before freezing, as appropriate for downstream applications.
    If the Alternate Protocol (FNA with negative pressure) is performed, the supernatant can still be saved for analysis, but the sensitivity will be markedly reduced due to the dilution introduced by the flushing step.
3 Returning to the cell pellets, gently resuspend the cells by pipetting using 500 µl R10 medium. Transfer all the resuspended cells into a single 15‐ml conical tube. Use 500 µl R10 medium to collect residual cells, and transfer this suspension into the same 15‐ml conical tube.
  The cell pellets from each wash can be merged to increase yield for downstream assays.
4 Centrifuge the 15‐ml conical tube containing all recovered cells for 10 min at 900 × *g*, 4°C.
5 Remove the supernatant without disturbing the cell pellet and dispose of appropriately. Gently resuspend the cell pellet in 2 ml R10 medium.
  We gently pour the supernatant into an appropriate liquid decontamination flask (e.g., containing 1% Virkon), but aspiration by vacuum or by pipetting also works. The key is to not disturb the cell pellet.
6 To remove contaminating red blood cells, perform a density‐based isolation using Ficoll (Fig. 4C‐H):
  The underlay technique described here is functionally equivalent to the more commonly used overlay method for isolation of PBMCs from peripheral blood, and an overlay can be used. However, we have found that the gravity‐aided underlay gives a crisper interface and thus improves cell recovery.
  - Transfer the resuspended cells to a 5‐ml polystyrene FACS tube.
  - Pour 3 ml Ficoll into a 50‐ml conical tube.
    This step creates a reservoir of Ficoll for easier pipetting in subsequent steps. More than 3 ml of Ficoll can be dispensed, but this is the minimum recommended volume to have sufficient liquid for easy pipetting.
  - Aspirate 1 ml Ficoll using a P1000 pipet.
  - Take a glass Pasteur pipet and visually inspect both ends to ensure that there are no signs of damage or breakage.
    As sub‐step “f” involves placing the operator's finger on the end of the Pasteur pipet, this visual check is critical to avoid the risk of sharps injury.
  - Place the Pasteur pipet into the 50‐ml conical tube and dispense the 1 ml Ficoll into the Pasteur pipet (Fig. 4C).
  - Immediately cover the top of the Pasteur pipet with your finger to create a seal. This will create a vacuum in the top of the pipet, which prevents the Ficoll from running out the bottom of the pipet (Fig. 4D and E).
  - With the Pasteur pipet sealed, transfer it to the 5‐ml FACS tube containing cells and gently insert the pipet into the tube until the tip touches the bottom of the tube.
  - Release your finger from the top of the Pasteur pipet. The Ficoll should begin to slowly drain from the Pasteur pipet into the tube and form a layer under the cell‐containing R10 medium (Fig. 4F). If the Ficoll does not drain, lift the Pasteur pipet a tiny amount (e.g., 1 mm) and the Ficoll will begin to drain.
  - Keeping the Pasteur pipet in the 5‐ml FACS tube, pipet an additional 1 ml Ficoll into the Pasteur pipet.
  - Once the Ficoll has drained to the narrow neck of the Pasteur pipet, it will stop draining further (Fig. 4G). Place your finger on the top of the pipet to seal it, carefully remove the pipet from the 5‐ml tube, and dispose of the pipet in an appropriate sharps bin.
  - Cap the 5‐ml FACS tube only to the “first position” and do not push completely down, as that risks disrupting the layering, especially when removing the cap.
    Given the small number of cells, using the small‐diameter/volume (i.e., 5‐ml) FACS tube minimizes cell loss.
    We have found that the specific dimensions (length and bore) of glass Pasteur pipets are superior for performing underlays as compared to those of plastic Pasteur pipets that we have tried. However, if a glass pipet is unsuitable (for example, for high‐containment work), a plastic Pasteur pipet can be used.
    If using a glass Pasteur pipet, it is critical to check it for damage before use to avoid cuts due to rough or broken edges.
7 Centrifuge the 5‐ml FACS tube containing the layered cells for 20 min at 900 × *g*, room temperature. Ensure that the centrifuge brake is turned off or this step.
*IMPORTANT*: It is critical that the brake is off on the centrifuge, as otherwise, when it begins to decelerate, the interface will be disturbed and cells will not be properly retained.
8 Using a P1000 pipet set to 1 ml, carefully collect the cells at the interface and transfer to a fresh 15‐ml conical tube (Fig. 4H). Repeat this step, collecting all material into the same 15‐ml conical tube.
  Doing the step twice collects a total of 2 ml of medium, which is essentially the entire volume of non‐Ficoll‐medium contents in the tube. The key is to gently pipet from the interface to collect as many cells as possible. Moving the pipet in a slow circular motion around the edges of the tube, while keeping the tip at the interface, will aid cell recovery.
9 Add 13 ml R10 medium to the 15‐ml conical tube containing the isolated cells to wash them.
10 Centrifuge the tube for 10 min at 900 × *g*, room temperature.
11 Remove and dispose of the supernatant, as appropriate, without disturbing the cell pellet.
  See the notes on step 5 for considerations of supernatant removal.
12 Resuspend the cells in 500 µl R10 medium.
13 Count the cells to determine number of cells recovered:
  - Add 90 µl trypan blue to a 1.5‐ml microcentrifuge tube.
  - Gently mix the cell pellet to homogenize.
  - Transfer 10 µl of cells into the trypan‐containing tube.
  - Gently pipet with a P200 set to 100 µl to mix the cells.
  - Take 10 µl and load onto a hemocytometer slide.
  - Count the cells and determine the average number of live cells in a 4 × 4 grid.
  - Determine the total number of cells as:
  [counted cells] × 5 × 10^4^ = total number of cells recovered from the FNA
  Training on the appropriate use of a hemocytometer is outside the scope of this protocol, but numerous online resources exist that provide guidance through the process.
  The specific formula for calculating the number of recovered cells is:
  [counted cells]
  × 10 (for 1:10 dilution)
  × 10,000 (to go from 0.1 µl counted to concentration per 1 ml)
  × 0.5 (for 0.5 ml resuspension volume)
  = total number of cells
  If the number of cells counted at a 1:10 dilution is too large or small due to specifics of cell recovery, the dilution factor should be adjusted and the cells re‐counted, with the above formula changed accordingly.
  Alternatively, an automated cell counter can be used for cell counting following the manufacturer's instructions. As the cell population isolated from the lymph node are all immune cells, settings optimized for counting of PBMCs are appropriate.
14 The cells are now ready for use. The appropriate number of cells can either be taken directly into the relevant assay (e.g., flow cytometry) or cryopreserved for future use.
15 If cells are to be cryopreserved, perform the following freezing protocol:
  - Centrifuge 5 min at 750 × *g*, room temperature.
  - Remove of and dispose of the supernatant, as appropriate, without disturbing the cell pellet.
    See the notes on step 5 for considerations of supernatant removal.
  - Resuspend the cells in 500 µl pure FBS and transfer to a fresh labeled cryovial.
  - Transfer cells to 4°C and allow to rest for 20 min.
  - Collect the cells from 4°C and add 500 µl of the 20% DMSO/FBS mixture.
    Do this as quickly as possible to minimize toxicity to the cells
  - Place the cryovial into the CoolCell container and immediately transfer to −80°C.
  - The following day, transfer cells to an appropriate storage box in either liquid N_2_ or −150°C freezer for long‐term storage and record in freezer logs, as appropriate.
    The cells can be left at −80°C over the weekend if samples are processed on a Friday.

## REAGENTS AND SOLUTIONS

### R10 medium

445 ml of RPMI‐1640 with l‐glutamine (ThermoFisher Scientific, cat. no. 22400089)
50 ml heat‐inactivated fetal bovine serum (FBS; multiple vendors)
5 ml penicillin/streptomycin (ThermoFisher Scientific, cat. no. 15140122)
Stored up to 2 weeks at 4°C
  Prepare medium in a tissue culture cabinet to maintain sterility.

## COMMENTARY

### Critical Parameters

The utility of lymph node (LN) fine‐needle aspiration (FNA), both with and without negative pressure, in revealing key insights into immune processes in the context of neuroimmune conditions (Al‐Diwani et al., 2022; Damato et al., 2022)**,** vaccination (Day et al., 2022; Law et al., 2022; Lederer et al., 2022; Mudd et al., 2022; Turner et al., 2020, 2021), and basic physiology (Provine et al., 2024) is well documented. To date, the sampling technique without negative pressure (Basic Protocol 1) as a means to study soluble LN‐associated proteins has been used exclusively for cervical lymph node sampling (Al‐Diwani et al., 2024; Provine et al., 2024), whereas vaccine studies have primarily utilized negative pressure during aspiration (Alternate Protocol). There are relative strengths to both approaches.

**Figure 5:**
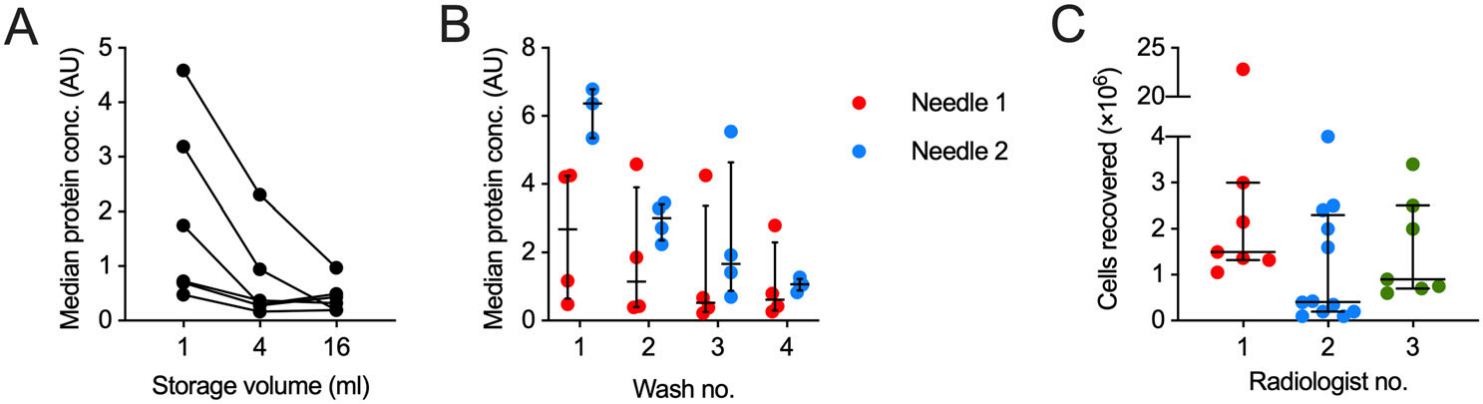
Protein and cell recovery considerations. (**A**) Median protein concentration recovered from LN FNA supernatants (*n* = 6) diluted to different volumes to model recovery of cells into different amounts of PBS. (**B**) Median protein concentration (*n* = 4; except needle 2 wash no. 1, *n* = 3) recovered from each needle wash of each of two FNA needle passes. Data in A and B were generated using the Olink assay as described in (Provine et al., 2024). (**C**) Number of cells recovered from CLN FNA by three different radiologists (*n* = 26). Median and interquartile range are shown in (**B**) and (**C**).

Scientifically, the primary consideration is what kind of sample material is the highest priority to collect: LN cells or LN fluid. The two protocols presented have relative trade‐offs with respect to lower cell yield but better recovery of soluble analytes (Basic Protocol 1) versus improved cell yield but dilution of soluble analytes (Alternate Protocol). Other studies have also reported similarly regarding improved cell recovery using negative pressure sampling (Havenar‐Daughton et al., 2020). Unsurprisingly, increased collection volume dilutes protein and reduces sensitivity (Fig. 5A). Therefore, the optimal technique should be selected based on the scientific question. Yield is improved in the second needle pass relative to the first (Fig. 5B). Although we have not analyzed protein recovery beyond two needle passes, cellular yields improve. The only trade‐off is the increased discomfort reported by participants with repeated sampling. For this reason, the Alternate Protocol includes the use of local anesthetic to improve tolerability. Overall, we have found high participant satisfaction with the technique (Fig. 1), which is an important factor if considering longitudinal or repeat sampling. Maintenance of participant satisfaction should be considered when weighing up the relative need for multiple needle passes.

Major practical considerations are operator‐to‐operator variability and sample recovery biases due to familiarity with the sampling technique (Fig. 5C). Operator‐to‐operator variability has also been noted elsewhere (Havenar‐Daughton et al., 2020). These factors should be considered during study design to avoid inadvertently biasing study outcomes.

### Troubleshooting Table

Possible problems, their causes, and proposed solutions are outlined in Table 1.

**Table 1:** Troubleshooting

**Problem**	**Possible cause**	**Solution**
Insufficient cell yield	FNA needle not fully in the LN. Insufficient sample collection time in the LN.	Extend corkscrew action during sample collection. Perform multiple collections (using fresh needle each time). Use Alternate Protocol.
	Accidental aspiration of cell pellet.	Take care to properly orient microcentrifuge tubes and aspirate away from cell pellet (Fig. 4A and B).
	Poor recovery of cell interface (Basic Protocol 2, steps 6 and 7).	Take additional care when forming the Ficoll layer to avoid mixing or disruption to the layer. Make sure the brake is off during centrifugation (step 7). During sample collection (step 8), pipet up extra material, including some of the Ficoll to guarantee all cells are recovered.
Insufficient soluble protein concentration	Excess dilution of protein during sample recovery (e.g., Alternate Protocol).	If detection of a specific soluble analyte is critical, Basic Protocol 1 is preferable.
	Merging of multiple washes or needle passes.	The first wash of the second needle gives the best protein recovery, and so should not be merged with other washes.

### Understanding Results

Using Basic Protocol 1, we performed cervical LN FNA on 26 individuals with a median cell recovery of 1.34 × 10^6^ lymph node cells (range 0.1 × 10^6^ to 22.8 × 10^6^ cells). Both operator (Fig. 5C) and participant health status (i.e., active neuroimmune disease) can affect cell recovery. Cell‐free lymph node protein recovery was reliable when using a 1‐ml wash volume, but larger collection volumes negatively affected sensitivity (Fig. 5A). Additionally, using a second needle had clear benefits for protein recovery (Fig. 5B). The desired cell number for downstream assays should thus be carefully considered, and if needed, the Alternate Protocol can be used to increase cell yield. However, as this uses a larger collection volume, it may affect the detection of specific proteins in the supernatant. The relative benefits of each procedure will need to be empirically determined case by case. Overall, the sampling technique was well tolerated and acceptable to participants (Fig. 1), which facilitates repeated longitudinal sampling.

### Time Considerations

The clinical FNA protocols (either Basic Protocol 1 or Alternate Protocol) take ∼2 hr to complete. Most of this time is devoted to procedure setup and patient welfare checks. The preparation of samples (Basic Protocol 2) takes ∼4 hr. There are several steps with fixed times (i.e., centrifugation), but intervening steps will be accomplished more quickly as familiarity with the protocol increases.

### Author Contributions

**Adam Al‐Diwani**: Conceptualization; data curation; investigation; methodology; writing—original draft; writing—review and editing. **Deepsha Agrawal**: Investigation; methodology; visualization; writing—review and editing. **Fintan Sheerin**: conceptualization; methodology; writing—review and editing. **Callum Board**: Investigation; writing—review and editing. **Sarosh Irani**: Conceptualization; methodology; writing—review and editing. **Katrina Pollock**: Conceptualization; investigation; methodology; writing—original draft; writing—review and editing. **Nicholas Provine**: Conceptualization; data curation; formal analysis; funding acquisition; investigation; project administration; writing—original draft; writing—review and editing.

### Conflict of Interest

N.M.P. has received consulting fees from Infinitopes, LLC. K.M.P. has received payment or honoraria from CSL Seqirus and Sanofi Pasteur, and has participated in data safety monitoring boards for Moderna.

## Data Availability

Data are available upon reasonable request to the corresponding authors.
